# Potential Role of the Gut Microbiome In Colorectal Cancer Progression

**DOI:** 10.3389/fimmu.2021.807648

**Published:** 2022-01-07

**Authors:** Jaeho Kim, Heung Kyu Lee

**Affiliations:** Graduate School of Medical Science and Engineering, Korea Advanced Institute of Science and Technology (KAIST), Daejeon, South Korea

**Keywords:** gut microbiota, colorectal cancer, immunotherapy, chemotherapy, immune checkpoint inhibitors

## Abstract

An increasing number of studies have revealed that the progression of colorectal cancer (CRC) is related to gut microbiome composition. Under normal conditions, the gut microbiome acts as a barrier to other pathogens or infections in the intestine and modulates inflammation by affecting the host immune system. These gut microbiota are not only related to the intestinal inflammation associated with tumorigenesis but also modulation of the anti-cancer immune response. Thus, they are associated with tumor progression and anti-cancer treatment efficacy. Studies have shown that the gut microbiota can be used as biomarkers to predict the effect of immunotherapy and improve the efficacy of immunotherapy in treating CRC through modulation. In this review, we discuss the role of the gut microbiome as revealed by recent studies of the growth and progression of CRC along with its synergistic effect with anti-cancer treatment modalities.

## Introduction

Colorectal cancer (CRC) is one of the most common types of cancer and is the third highest leading cause of death worldwide (1). Numerous epidemiological studies have demonstrated that the prevalence of CRC is related to a western diet and intake of dietary fiber, thus highlighting the important relationship between diet and CRC (2–5). In this context, the gut environment, including the microbiome, has been in the spotlight and has emerged as an important factor related to CRC (6).

A multitude of microorganisms live in the intestines of mammals. In the human intestine, there are more than 1000 species and 10^14^ microorganisms forming a colony (7). They play an important role in maintaining a normal physiologic environment, including energy metabolism, interacting with the normal gut barrier system, promoting the survival of epithelial cells, and, importantly, protecting our body against other external or opportunistic pathogens (8). Over the past few decades, studies have shown that the gut microbiome influences the host significantly (9–11). Dysbiosis in the intestines is known to be associated with the pathogenesis of a variety of diseases, including neurological, gastrointestinal, and metabolic diseases (12). Changes in the gut microbiome can be induced by eating habits or changes in environmental factors and studies have shown that changes in the gut microbiome induce CRC through inflammatory diseases, microbial metabolites, or virulence factors (13–15). The gut microbiome has been demonstrated to affect not only the generation of CRC, but also its progression. Furthermore, the gut microbiome has been associated with controlling the efficacy of cancer treatment and the toxicities of therapeutic agents. Thus, therapeutic agents, such as probiotics, that can control the gut microbiome are expected to among the most effective approaches for helping to fight CRC (16, 17).

Recent advances in our understanding of the role of the gut microbiome are due to the development of technologies, such as 16S rRNA sequencing, that enable the discovery of many microorganisms in the intestine that could not be identified previously (18). Many studies related to metabolomics and metagenomics describe the effects of these gut microbes on the human body, and some studies revealed their involvement in cancer prevention, tumorigenesis, and anti-cancer effects (19, 20). In particular, changes in gut microbial metabolites, such as short-chain fatty acids (SCFAs), polyphenols, vitamins, tryptophan catabolites, and polyamines produced or affected by the gut microbiota, may have a wide range of effects on the formation and progression of CRC and even metastasis (21). As our understanding of the role of the anti-cancer immune response in the tumor microenvironment during cancer progression and treatment increases, the effect of the gut microbiome on tumor immunity is also receiving greater attention (21). It is known that changes in the gut microbiota not only affect tumor immunotherapy, but also affect therapeutic toxicity (22). Thus, modulation of the gut microbiome can be used as a novel treatment modality.

The gut microbiome has emerged as an important factor in various diseases, and the relationship between the gut microbiome and CRC has become an important issue in several studies. In this review, the potential role of the gut microbiome will be reviewed with a focus on how the gut microbiome affects the tumorigenesis processes associated with CRC. Furthermore, we discuss methods of gut microbiome modulation that can be used to treat CRC.

## Correlation Between CRC and Gut Microbiome

With changes in western dietary habits worldwide, the incidence of CRC is expected to increase steadily, resulting in 2.2 million new cases by 2030 (23). Studies have shown that approximately 90% of CRC occurs sporadically and the remainder is caused by genetic factors or exposure to specific environmental factors (24–27). In particular, lifestyle factors such as physical inactivity, smoking history, western diet, low fiber intake, alcohol intake, and obesity are major influences on CRC. It is important to note that most of these environmental factors can induce changes in the gut microbiota (26, 28, 29). Many studies have confirmed that susceptibility to CRC or tumor progression is affected by changes in the gut microbiome, which has been found to induce inflammation, DNA damage, or metabolites produced from microorganisms (30).

Evidence from several studies has suggested the existence of a close link between the gut microbiome and the host during the development of CRC (31–33). Studies using high-throughput microbiome sequencing have been conducted to investigate the microbiome community in tumor-formed and normal colon tissues (27), enabling a better understanding of the differences in gut microbiome between CRC and healthy patients. Reports have shown that the diversity and richness of the gut microbiome decreases in CRC patients (33, 34). In particular, analysis of the gut microbiome of CRC patients revealed that significant changes in specific microbial groups occurs, leading researchers to hypothesize that these changes might have a greater impact on the mucosal immune response of CRC patients compared to that of healthy individuals (34). A total of 11 operational taxonomic units (OTUs) belonging to the genera *Enterococcus*, *Escherichia*/*Shigella*, *Klebsiella*, *Streptococcus*, and *Peptostreptococcus* were significantly found to be more abundant in the gut microbiota of CRC patients, while 5 OTUs belonging to *Roseburia* and other butyrate-producing bacteria from the *Lachnospiraceae* family were less abundant (35). In addition, dysbiosis was observed in the gut microbiome of CRC patients as the balance between microorganisms was disrupted (36). Dysbiosis of gut microbiota and increased intestinal permeability induce colonic inflammation and may cause the promotion or progression of CRC (37). *Fusobacterium nucleatum* (*F. nucleatum*) is significantly increased in human CRC compared to healthy patients (38). Moreover, early-stage CRC patients (advanced adenoma) have a different microbiome composition compared to advanced-stage CRC patients (definitive CRC) (35, 39). These studies indicate a very close correlation between CRC and the gut microbiome; however, further investigation is still required to fully elucidate the effect of the gut microbiome on CRC.

## Influence of the Gut Microbiome on CRC Formation

Although much is still unknown about the formation of CRC, chronic inflammation has been implicated in the initiation of malignancy. It is estimated that approximately 20% of malignant tumors occurring in the colon are preceded by chronic inflammation (40). During carcinogenesis, inflammatory cytokines and chemokines produced by cancerous cells attract immature myeloid cells or pro-inflammatory helper T cells. This pro-tumorigenic microenvironment is characterized by the synthesis of growth and angiogenic factors, as well as tissue remodeling enzymes, and the suppression of anti-tumor T-cell responses, favoring tumor progression (41).

Knowledge that the gut microbiome affects CRC formation was first obtained in the early 1970s. When the colon was exposed to a carcinogen called 1,2-dimethlylhydrazine in a germ-free mouse model, the degree of CRC formation was found to be significantly reduced (42). At the time, it was not possible to specify which bacteria caused this phenomenon. However, a similar experiment using various colon cancer models confirmed that the presence or absence of intestinal microbes had a significant effect on the formation of colon cancer (43, 44). Since then, many studies using high-throughput microbiome sequencing have identified the various intestinal microorganisms that affect CRC formation.

*Streptococcus bovis* (*S. bovis*) has been reported as one of the risk factors for CRC (45–47). *S. bovis* is normally colonized in the gastrointestinal tract. Thus, the occurrence of *S. bovis*-induced endocarditis or bacteremia was an early clue to its involvement in colon cancer (45). The association between inflammation and colon carcinogenesis was confirmed when the relationship between the pro-inflammatory potential of *S. bovis* proteins and their carcinogenic properties was observed (48, 49). *S. bovis* has been found to play an active role in CRC development, perhaps through an inflammation-based sequence of tumor development or propagation involving interleukin (IL)-1, cyclooxygenase-2 (COX-2), and IL-8 (48).

*F. nucleatum* is one of the most widely known strains related to CRC tumor formation (50). Metagenomic analysis showed that the commensal *Fusobacterium* spp. are associated with CRC in humans; however, it remains unclear whether this is indirect or causal (38). Castellarin and coworkers confirmed that the transcripts of the strain were increased approximately 400 times in CRC tumor tissue compared to normal tissue (50). In a study using the adenomatous polyposis coli (*APC) ^+/-^* mouse CRC model, *F. nucleatum* developed a pro-inflammatory environment which induced neoplasia progression in intestinal epithelial cells and recruited tumor-infiltrating immune cells (38). In addition, studies demonstrated that IL-17a was highly expressed in CRC patients with abundant *F. nucleatum* (51). This strain induces early carcinogenesis through increased bacterial adherence in the mucosal surface (52). *F. nucleatum* produces a unique protein called Fusobacterium adhesin A (FadA), which induces activation of the β-catenin signaling pathway after binding to E-cadherin, which is a potent oncogenic stimulator.

*Enterococcus faecalis* (*E. faecalis*) is a gut commensal bacterium that produces a superoxide from the autoxidation of membrane-associated demethylmenaquinone (53). Infection with *E. faecalis* induces DNA damage to intestinal epithelial cells by forming the superoxide. Thus, the abundance of *E. faecalis* was shown to be significantly increased in CRC patients compared to healthy individuals (35, 54, 55). Moreover, *in vitro* and *in vivo* studies demonstrated that *E. faecalis* can produce hydroxyl radicals (56, 57), which are potent mutagens that cause DNA breaks, point mutations, and protein-DNA crosslinking, thereby contributing to chromosomal instability and CRC risk (58).

Enterotoxigenic *Bacteroides fragilis* (*ETBF*) is a bacterium that produces *B. fragilis* toxin (BFT) and causes diarrhea and inflammatory bowel disease (IBD) (59–62). This strain plays a role in promoting tumors by elevating signal transducer and activator of transcription 3 (STAT3) and the Th17 response during colon tumorigenesis (60). Although STAT3 activation is required for colon tumorigenesis, it alone is not sufficient to trigger colon tumorigenesis by *ETBF.* Notably, IL-17-dependent nuclear factor kappa B (NF-κB) activation results in the formation of a proximal to distal mucosal gradient of CXC chemokines, which mediates the recruitment of CXCR2-expressing polymorphonuclear immature myeloid cells to cause *ETBF*-mediated distal colon tumorigenesis in parallel (62).

*Peptostreptococcus anaerobius* (*P. anaerobius*) induces a pro-inflammatory immune microenvironment and promotes tumor formation in the intestine. This strain plays a role in tumor formation by increasing the expression of pro-inflammatory cytokines in a mouse model and recruiting tumor-infiltrating immune cells such as immunosuppressive myeloid-derived suppressor cells (63). *P. anaerobius* increases the levels of reactive oxidative species that interact with toll-like receptor (TLR) 2 and TLR4 in colon cells to promote cholesterol synthesis and cell proliferation, ultimately causing dysplasia of colon cells (64).

Salmonella infections and colonization can be chronic and increase the risk of chronic cholecystitis and other gastrointestinal diseases, including cancers (65). Salmonella promotes colon tumorigenesis by relying on AvrA protein, which can activate both the Wnt/b-catenin and STAT3 signaling pathways in colon tumor cells (66–68). Salmonella also produces a genotoxin called typhoid toxin, which damages DNA *via* the phosphoinositide 3-kinase (PI3K) pathway in colonic epithelial cells (69). The reduced DNA repair capacity and inability to activate appropriate checkpoint responses have been associated with increased genomic instability in APC-deficient cells exposed to genotoxin. *Campylobacter jejuni* produces a cytolethal distending toxin (CDT), a genetic toxin with DNAse activity that causes DNA double-strand breaks and promotes colorectal tumorigenesis (70). Rapamycin, which inhibits mammalian target of mTOR signaling in mammals, has been shown to attenuate *C. jejuni*-induced colitis and carcinogenesis (70, 71).

Sulfate-reducing bacteria (SRB) are a microbiome component that is of particular interest with respect to colitis (72). These microorganisms can produce hydrogen sulfide (H_2_S) by using methionine and cysteine as substrates. Studies have shown increased amounts of SRB in the stool of CRC patients compared to healthy individuals (73). H_2_S produced by SRB can stimulate CRC progression by inhibiting butyrate oxidation and destroying the gut barrier, as well as induce DNA damage through reactive oxygen species (ROS) (74, 75).

Research to understand the relationship between other intestinal microbes with CRC formation is still ongoing. Thus, the bacteria discussed above do not constitute all of the causative bacteria of CRC.

## Influence of Gut the Microbiome on CRC Progression

**Figure 1:**
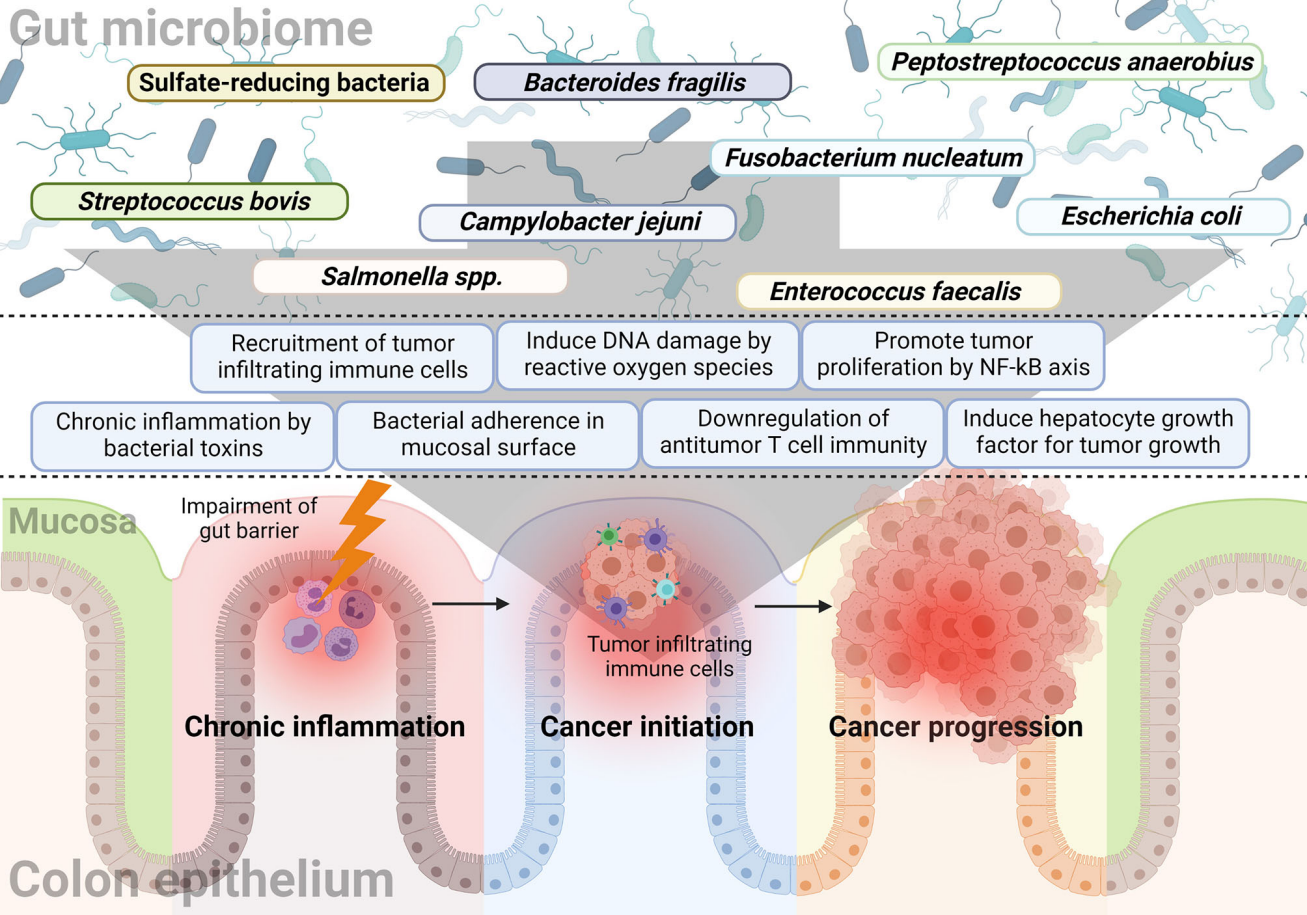
The relationship between the gut microbiome and sequential progression of colorectal carcinoma. Specific gut microorganisms induce chronic inflammation in the colorectal epithelium. For example, typhoid toxin or colibactin secreted by *Salmonella* or *E. coli*, respectively, leads to pro-inflammatory cytokine production and bacterial adherence. Chronic inflammation is one of the major causes of CRC and increased ROS with epithelial cell DNA damage also play a major role in cancer initiation by the gut microbiome. Some microorganisms like *F. nucleatum* and *B. fragilis* induce a tumor-favorable immune microenvironment by reducing CD3+ T cell density along with the recruitment and proliferation of CD4+CCR6+IL17A+ Th17 cells. Furthermore, bacterial components such as putative cell wall binding repeat 2 surface protein in *P. anaerobius* activate the NF-κB signaling pathway in CRC tumor cells and promote tumor cell proliferation. Colibactin-producing *E. coli* encodes enzymes responsible for HGF synthesis and induces senescence and tumor growth.

The gut microbiome affects not only the formation of colon malignancy, but also its progression. Published literature related to the development of CRC has demonstrated that many bacteria affect tumor development and growth. In addition, it was observed that the progression of colon adenoma was promoted in a spontaneous CRC mouse model characterized by expression of mutated *Apc*, a tumor suppressor gene (76). This section will describe research findings associated with progression-related mechanisms rather than tumor formation. **Figure 1** summarizes the bacteria and their mechanisms of involvement in CRC initiation and progression.

The presence of *F. nucleatum* is associated with worse prognosis in CRC patients (77, 78). Expression of tumor necrosis factor alpha, β-catenin, and NF-κB was increased in the *F. nucleatum*-abundant group and COX-2, matrix metallopeptidase 9, and NF-κB were highly expressed in the *B. fragilis*-abundance group. Immunohistochemical analysis showed that Kirsten rat sarcoma virus (KRAS) and proto-oncogene B-Raf (BRAF) expression were increased in the presence of *F. nucleatum* and *B. fragilis* (78). *F. nucleatum*-high cases were inversely associated with the density of CD3+ T cells (79). Experimental evidence suggests that *F. nucleatum* can promote colonic tumor development by downregulation of anti-tumor T cell-mediated adaptive immunity. Natural killer cells (NK cells) were also found to be affected by *F. nucleatum* in various carcinomas including CRC (80). Gur and colleagues found that the Fap2 protein of *F. nucleatum* directly interacts with T cell immunoreceptor with Ig and ITIM domains (TIGIT) to inhibit the cytotoxicity of NK cells.

*ETBF* was also revealed to support the progression of malignancy as well as tumorigenesis (81). This strain induces the secretion of exosome-like nanoparticles in intestinal epithelial cell lines and contains chemokine CC motif ligand 20 and prostaglandin E2 in the particle. Thus, *ETBF* induces the recruitment and proliferation of CD4+CCR6+IL17A+ Th17 cells *via* the IL-17 signaling pathway, thereby participating in tumorigenesis and cancer cell growth.

Long, et al. found that the surface protein of *P. anaerobius*, putative cell wall binding repeat 2 (PCWBR2), promotes CRC development in *APC^+/-^* mice (63). PCWBR2 initiates the oncogenic PI3K-Akt signaling pathway that directly binds to the intestinal epithelial cell receptor integrin α2/β1 and promotes tumor cell proliferation *via* the PCWBR2-integrin α2/β1-PI3K-Akt-NF-κB signaling axis.

*Escherichia coli* (*E. coli*), which is the most highly abundant strain residing in the intestine, is also closely related to the growth of CRC. Studies have shown that the level of mucosal-associated *E. coli* is increased in CRC tumor tissues compared with in normal colon tissues (82). The pathogenic *E. coli* strain showed a correlation with inflammation and ROS production, which may propagate tumor infiltration (83). *E. coli* has polyketide synthase which codes for production of colibactin, the polyketide-peptide genotoxin found to play a significant impact on tumor growth (84, 85). In a xenograft model, colibactin-producing *E. coli* indirectly promotes tumor growth by inducing hepatocyte growth factor (HGF) (86). HGF is the main mechanical link between pks+ (which encodes enzymes responsible for HGF synthesis) *E. coli*-induced senescence and tumor growth. Other factors, including microRNA-20a-5p, sentrin-specific protease 1 (SENP1), and activated HGF receptors, are also affected by the presence of pks+ *E. coli* in human CRC.

In contrast, the presence or enrichment of certain intestinal strains leads to anti-cancer effects on the growth of CRC. Numerous animal studies have shown several emerging chemical candidates as key mechanisms for probiotics to induce protective effects against CRC. *Faecalibacterium prausnitzii* is a potential probiotic that can downregulate the NF-kB pathway in gut epithelial cells by producing hydrophobic microbial anti-inflammatory molecules and prevent colitis in animal models (87). *Lactobacillus rhamnosus* GG and *Bifidobacterium lactis* Bb12 help to prevent abnormal epithelial proliferation in patients with a history of polyps and improve the intestinal epithelial tight junction barrier (88). *Lactobacilli* and *Bifidobacteria* were suggested to play a role in suppressing tumor progression and volume in a CRC mouse model (89, 90). The presence of these probiotics was confirmed to induce increasing SCFA production, thus inducing apoptosis and inhibiting tumor proliferation (91). Butyrate, one of the SCFA metabolites produced by probiotics, can induce the expansion of T reg lymphocytes for regulating the immune response in colorectal tissues and suppressing carcinogenesis and tumor growth (92).

## Influence of the Gut Microbiome on CRC Treatment

**Figure 2:**
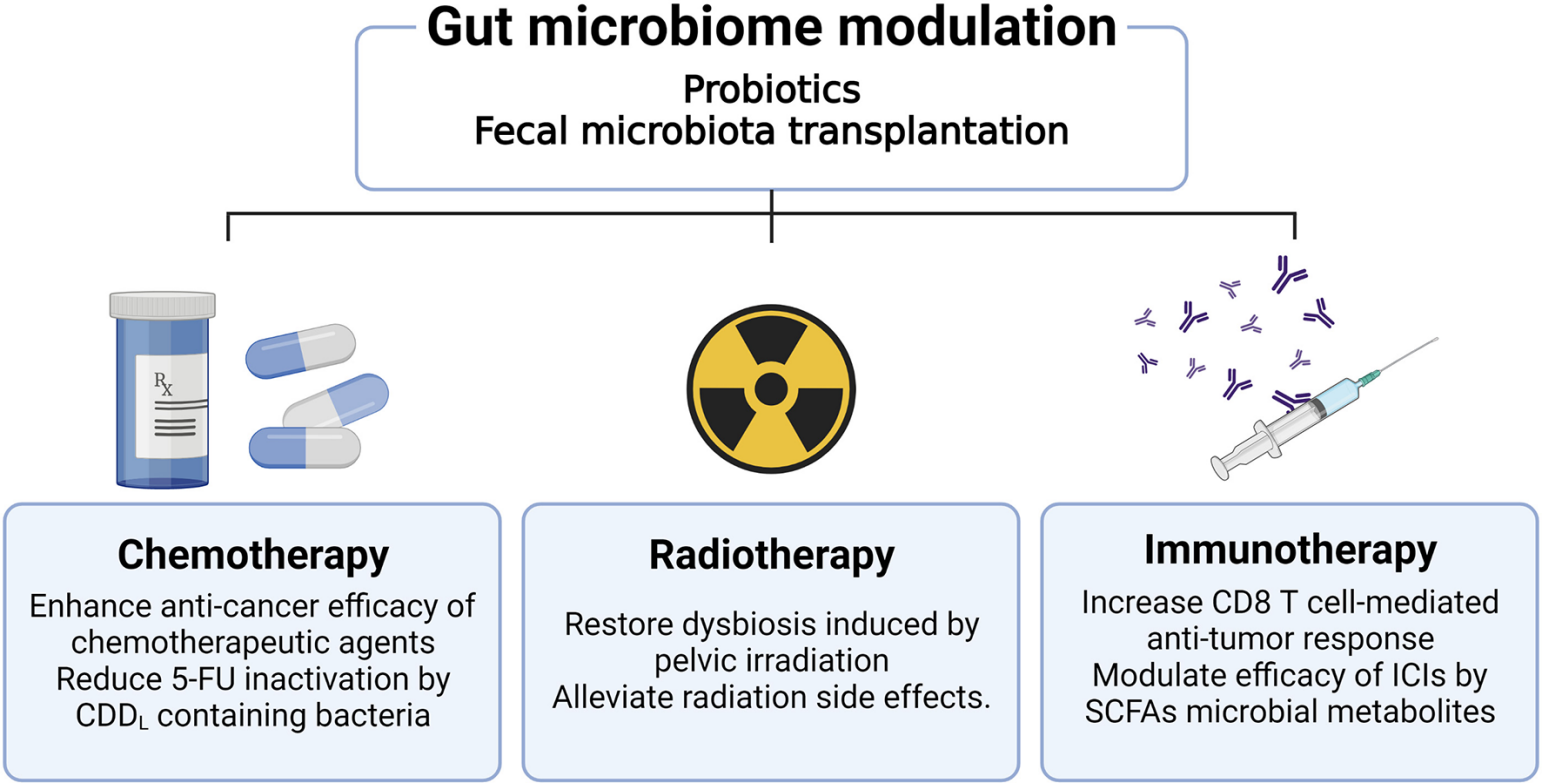
Effects of gut microbiome modulation on cancer treatment. Therapies which modulate the gut microbiome, including administration of probiotics or fecal microbiota transplantation, improve the efficacy of cancer treatment. Administration of antibiotics can reduce the efficacy of oxaliplatin and CpG oligodeoxynucleotides chemotherapeutic agents. The use of antibiotics increases pathogenic bacteria such as *Escherichia shigella* and *Enterobacter*, as well as reduces the anti-cancer effect of 5-FU. Radiation of the pelvic area causes dysbiosis and has the potential to affect the treatment modality of CRC. Furthermore, radiation-induced gut epithelial damage worsens the prognosis of CRC patients. These radiation side effects can be ameliorated through fecal microbiome transplantation as well as probiotics administration. The gut microbiota plays a role in modulating mucosal immunity in the colorectal region, acting to improve the efficacy of immunotherapy by enhancing the CD8+ T cell immune response or SCFA metabolite production.

Because the gut microbiome has been closely associated with CRC, numerous studies have been focused on investigating its effect on CRC treatment. Research related to the effect of gut microbiome on tumor treatment is the most important part of the cancer-microbiome research field and many studies are being conducted in combination with various treatment modalities to apply it to clinical cancer treatment. In addition to existing chemotherapeutic agents or radiotherapy, new discoveries are being made about the synergistic effects of the gut microbiome with immune checkpoint inhibitors (ICIs) (93). **Figure 2** summarizes the research findings discussed below.

## Chemotherapy

The gut microbiota can modulate the efficacy of conventional chemotherapy. For example, it is known that certain gut microbiota may play a role in regulating cytotoxicity by participating in the metabolic process of anti-cancer drugs. The anti-cancer effect of platinum-based chemotherapeutic agents such as oxaliplatin and CpG oligodeoxynucleotides was decreased in mice treated with antibiotics (94), which exhibited lower cytokine secretion and ROS production, resulting in reduced tumor necrosis following anti-tumor therapy in the MC38 mouse colon tumor transplant model.

Gemcitabine has been shown to convert into an inactivated form with reduced anti-cancer effect when a specific gammaproteobacteria is present in the tumor (95). Gammaproteobacteria contain a long isoform of the cytidine deaminase enzyme which converts gemcitabine into an inactivated form. The anti-cancer effect was shown to be suppressed when the bacteria were eliminated by antibiotic treatment in a mouse model of CRC (95). Even in mouse tumor experiments using 5-fluorouracil (5-FU), antibiotic administration reduced the anti-cancer effect of 5-FU administration in the CRC model (96). In 16S rRNA seq analysis, pathogenic bacteria such as *Escherichia shigella* and *Enterobacter* were significantly increased when antibiotics were administered, and these changes were restored by taking probiotics.

*F. nucleatum*, which was previously known to greatly influence tumor initiation and progression, affects CRC treatment outcomes as well as CRC risk and dysplasia. A qPCR analysis based on colorectal tissue samples from 122 CRC patients confirmed a better prognosis occurred in patients with low *F. nucleatum* levels (77, 97). The level of *F. nucleatum* enrichment was positively correlated with poor response to 5-FU and oxalipatin in CRC patients (98). *F. nucleatum* stimulates the TLR4 and Myd88 innate immune signals and interferes with apoptosis, contributing to activation of the autophagy pathway and CRC chemoresistance (98).

## Radiotherapy

Dysbiosis caused by radiation therapy has the potential to adversely affect the other treatment modalities of CRC. Analysis of the gut microbiome after radiation treatment showed a decrease in commensal bacteria such as *Bifidobacterium*, *Faecalibacterium*, and *Clostridium* spp., as well as an increase in *Bacteroides* and *Enterococcus* spp (99). In addition, in the case of patients receiving radiation therapy to the pelvic region, there was a tendency for the *Fusobacteria* taxa to increase by about 3% (100). These changes show the potential for tumor-promoting capacities. These microbiota can pass through the impaired gut barrier as a result of epithelial inflammatory damage caused by radiation therapy, leading to an additional intestinal inflammatory response and tissue damage (101).

## Immunotherapy

Certain intestinal microbes are involved in tumor growth by regulating the immune response. Studies have been conducted to elucidate the mechanism of intestinal microbes and how they affect the efficacy of immunotherapeutic agents. In 2015, it was reported that the commensal gut microbiome could enhance the anti-tumor efficacy of programmed death-ligand 1 (PD-L1) ICIs through two mouse studies. Cytotoxic T-lymphocyte-associated protein 4 (CTLA-4) inhibitors are one of the most widely used ICIs in clinical practice. The efficacy of CTLA-4 inhibitors was demonstrated to be altered by the population of the gut microbiome (102). The literature has identified an important role for *Bacteroides* species in the immunostimulatory modulation of CTLA-4 blockade. The modulation of ICI efficacy mediated by bacterial species in the gut microbiome is not limited to CTLA-4 signaling. The efficacy of a PD-L1 inhibitor was also shown to be modulated by the gut microbiota composition in a mouse tumor model (103). Recent studies have indicated that the anti-tumor effect was found associated with various bacteria such as *Akkermansia*, *Faecalibacterium*, *Clostridiales*, and *Bifidobacterium* spp (104–106). Although some details remain to be understood, this anti-tumor effect has been partially attributed to SCFA microbial metabolites such as butyrate and propionate (107). Another mechanism for modulation of ICIs is that host immune cells can interact directly with specific bacteria. *Akkermansia muciniphila* improves the efficacy of immunotherapeutic agents in an IL-12-dependent manner through direct interaction with dendritic cells in the lymph node (106). *Bacteroides* spp. can also directly increase Th1 and CD8 T cell anti-tumor immune responses (102).

## Microbiome Modulation for Colon Cancer Treatment

Growing evidence clearly illustrates the significant influence that the gut microbiome has on tumors. Thus, it is not surprising that attempts have been made to inhibit tumor growth or modulate the efficacy of tumor therapy by regulating the gut microbiota. Efforts are ongoing to increase the effectiveness of tumor treatment and reduce side effects through fecal microbiome transplantation as well as probiotic therapy.

We discussed results from studies showing that *Lactobacilli* and *Bifidobacterium* affect the occurrence and progression of CRC in animal models (89–91). Some probiotics can help to not only enhance the effects of anti-cancer therapeutic agents but also alleviate the side effects caused by conventional cancer treatments (108). However, these probiotics also have the potential to act as opportunistic pathogens that can easily penetrate the intestinal barrier and immune environment after weakening by intestinal tumors (109). Appropriate probiotics with appropriate administration methods that can enhance anti-cancer effects and alleviate side effects are needed.

Fecal microbiota transplantation (FMT) is an emerging biotherapeutic method for altering the microbiota by transplanting stool information from healthy donors to patients (110). FMT can be applied to various gastrointestinal diseases including *C. difficile* infection, IBD, and restored eubiosis (111, 112). Many efforts are being made to apply FMT in the clinic as a tumor treatment. Reports have shown that FMT could be used to overcome resistance to immunosuppressants in the CRC mouse model (113). In addition, FMT can be helpful in alleviating the side effects of ICIs such as immune checkpoint inhibitor-associated colitis (114). Complete resolution of colitis through FMT was sustained for 53 days after one dose and for 78 days after two doses. Although clinical application as a treatment for CRC is still unexplored, a recent FMT study of melanoma patients reported that FMT succeeded in overcoming resistance to immunotherapy in patients who did not respond to immunotherapy (115, 116). These results suggest that FMT can be effectively used in the treatment of CRC. However, since the gut microbiome environment consists of a very large network with many unknowns remaining, more research is needed before microbiome modulation can be administered as an anti-cancer treatment in CRC.

## Conclusion

Various animal and clinical experiments have demonstrated that changes in the composition of the gut microbiota affect the initiation of precancerous cancer lesions and cancer progression. Because the colorectal region is a site where changes in the gut microbiota can influence the organs directly, CRC is considered to be affected by the gut microbiome more than other tumors. Studies of the gut microbiome revealed that dysbiosis occurred more frequently in CRC patients than in healthy people. The proportion of butyrate-producing bacteria was found to be reduced along with inflammation in the intestine while opportunistic pathogens were increased. Epidemiological studies have highlighted dietary factors such as western eating habits and reduced dietary fiber intake as risk factors for CRC, suggesting the gut microbiome as one of the mechanisms linking these factors to CRC. Dietary fiber can be fermented into SCFAs by intestinal bacteria and animal experiments demonstrated that various SCFAs such as butyrate could affect cancer initiation and progression. Finally, the use of antibiotics may also be a risk factor for CRC and studies of the gut microbiome demonstrate its involvement in this effect.

Many published results have demonstrated that the gut microbiome acts as an important key factor in the initiation and progression of carcinoma in the treatment of CRC. However, we still understand only a small part of the gut microbiome and further research is needed to elucidate the underlying mechanisms and to modulate the gut microbiome as an important strategy in the treatment and prevention of CRC. This review describes the gut microbiome strains that affect each stage of the tumorigenesis process, including the underlying mechanisms, supplying an overview of the microbiota species likely involved in future studies examining the associations between the gut microbiome and CRC.

## Author Contributions

JK and HL wrote the manuscript. All authors contributed to the article and approved the submitted version.

## Funding

This study was supported by the National Research Foundation of Korea (NRF-2021M3A9D3026428 and NRF-2021M3A9H3015688) funded by the Ministry of Science and ICT of Korea.

## Conflict of Interest

The authors declare that the research was conducted in the absence of any commercial or financial relationships that could be construed as a potential conflict of interest.

## Publisher’s Note

All claims expressed in this article are solely those of the authors and do not necessarily represent those of their affiliated organizations, or those of the publisher, the editors and the reviewers. Any product that may be evaluated in this article, or claim that may be made by its manufacturer, is not guaranteed or endorsed by the publisher.
